# PET/CT imaging of spinal inflammation and microcalcification in patients with low back pain: A pilot study on the quantification by artificial intelligence‐based segmentation

**DOI:** 10.1111/cpf.12751

**Published:** 2022-04-01

**Authors:** Reza Piri, Amalie H. Nøddeskou‐Fink, Oke Gerke, Måns Larsson, Lars Edenbrandt, Olof Enqvist, Poul‐Flemming Høilund‐Carlsen, Mette J. Stochkendahl

**Affiliations:** ^1^ Department of Nuclear Medicine Odense University Hospital Odense Denmark; ^2^ Department of Clinical Research University of Southern Denmark Odense Denmark; ^3^ Eigenvision AB Malmö Sweden; ^4^ Department of Molecular and Clinical Medicine, Sahlgrenska Academy University of Gothenburg Gothenburg Sweden; ^5^ Department of Clinical Physiology Region Västra Götaland, Sahlgrenska University Hospital Gothenburg Sweden; ^6^ Department of Electrical Engineering Chalmers University of Technology Gothenburg Sweden; ^7^ Department of Sports Science and Clinical Biomechanics University of Southern Denmark Odense Denmark; ^8^ Chiropractic Knowledge Hub Odense Denmark

**Keywords:** fluorodeoxyglucose, low back pain, lumbar vertebrae, positron emission tomography, sodium fluoride

## Abstract

**Background:**

Current imaging modalities are often incapable of identifying nociceptive sources of low back pain (LBP). We aimed to characterize these by means of positron emission tomography/computed tomography (PET/CT) of the lumbar spine region applying tracers ^18^F‐fluorodeoxyglucose (FDG) and ^18^F‐sodium fluoride (NaF) targeting inflammation and active microcalcification, respectively.

**Methods:**

Using artificial intelligence (AI)‐based quantification, we compared PET findings in two sex‐ and age‐matched groups, a case group of seven males and five females, mean age 45 ± 14 years, with ongoing LBP and a similar control group of 12 pain‐free individuals. PET/CT scans were segmented into three distinct volumes of interest (VOIs): lumbar vertebral bodies, facet joints and intervertebral discs. Maximum, mean and total standardized uptake values (SUVmax, SUVmean and SUVtotal) for FDG and NaF uptake in the 3 VOIs were measured and compared between groups. Holm–Bonferroni correction was applied to adjust for multiple testing.

**Results:**

FDG uptake was slightly higher in most locations of the LBP group including higher SUVmean in the intervertebral discs (0.96 ± 0.34 vs. 0.69 ± 0.15). All NaF uptake values were higher in cases, including higher SUVmax in the intervertebral discs (11.63 ± 3.29 vs. 9.45 ± 1.32) and facet joints (14.98 ± 6.55 vs. 10.60 ± 2.97).

**Conclusion:**

Observed intergroup differences suggest acute inflammation and microcalcification as possible nociceptive causes of LBP. AI‐based quantification of relevant lumbar VOIs in PET/CT scans of LBP patients and controls appears to be feasible. These promising, early findings warrant further investigation and confirmation.

## 1 INTRODUCTION

Low back pain (LBP) is a very common symptom experienced by people of all ages. The global point prevalence is ~7% and 50%–80% of people will experience LBP at some point in their lives. In 90% of cases, the nociceptive source of pain is unknown and the LBP is classified as nonspecific (Koes et al., 2006; Maher et al., 2017). For these cases, no clinical test has yet been found able to accurately detect the specific tissue or morphological structure from where the noxious stimulation stems (Hancock et al., 2007; Maas et al., 2015). With modalities such as conventional radiography, computed tomography (CT) and magnetic resonance imaging (MRI), it is possible to identify various abnormalities in the lumbar spine. However, most of these are of limited or unclear clinical relevance for spinal pain, as the findings are detected frequently in individuals without LBP as well (Kasch et al., 2019; Steffens et al., 2014). Therefore, their relevance in identifying the nociceptive source of pain is controversial (Brinjikji, Diehn, et al., 2015). The vertebral endplates, intervertebral discs and facet joints have long been subject to scrutiny when it comes to localizing the nociceptive source of LBP. These structures are innervated and have been shown to produce pain when stimulated (Hancock et al., 2007; Maas et al., 2015). However, as implied, there are no widely accepted reference standards for identification of individuals in whom these structures contribute to their pain (Hancock et al., 2007; Kalichman et al., 2008; Maas et al., 2015).

Studies in the lumbar region and of the temporomandibular joint suggest that some cases of LBP and joint pain in general could potentially be due to mechanical overload (Huang et al., 2018; Wang et al., 2012). This will initially lead to acute and short‐term pain, but concomitantly give rise to a cascade of changes driven by inflammation and leading to degenerative changes and bone remodelling. In the initial stages, these changes are not detectable by the aforementioned imaging modalities. With hybrid imaging modalities, however, especially positron emission tomography/computed tomography (PET/CT), it may be possible to visualize and quantify early metabolic processes alongside later occurring morphologic derangements. The common PET tracer, ^18^F‐fluorodeoxyglucose (FDG), is used in PET/CT to trace inflammation in all parts of the body. The metabolism of inflammatory cells is high and gives rise to focally increased FDG uptake reflecting regional inflammatory processes including inflammation in LBP (Glaudemans et al., 2013; Hess et al., 2014). Another commonly used tracer is ^18^F‐sodium fluoride (NaF), which locates osseous changes in the body by exchange of fluoride ions with hydroxyl groups in hydroxyapatite. Tracer uptake reflects osteoblastic activity and bone perfusion and allows quantification of bone turnover (Blau et al., 1972; Chaudhari et al., 2021; Derlin et al., 2011; Park et al., 2021; Raynor et al., 2016). In this explorative, proof‐of‐concept case–control study, we compared inflammation and microcalcification using an artificial intelligence (AI)‐based bone segmentation method in different low back regions (vertebral bodies, facet joints and intervertebral discs) of subjects with and without LBP by means of FDG‐ and NaF‐PET/CT imaging.

## 2 METHODS

### 2.1 Study design

Subjects were selected as part of a prospective study ‘Cardiovascular Molecular Calcification Assessed by ^18^F‐NaF PET/CT’ (CAMONA), which was conducted in Odense University Hospital from 2012 to 2014 (Blomberg, Thomassen, Takx, Hildebrandt, et al., 2014; Blomberg, Thomassen, Takx, Vilstrup, et al., 2014; Piri et al., 2021). In the CAMONA study, 89 healthy volunteers and 50 patients with angina pectoris with an increased risk of cardiovascular disease were studied using FDG‐ and NaF‐PET/CT. Twelve individuals with LBP and age‐ and gender‐matched control individuals without LBP were selected to compare FDG and NaF uptake in the following low back regions: 12 individuals with LBP and age‐ and gender‐matched control individuals without LBP were selected to compare FDG and NaF uptake in the following low back regions; the L1–L5 lumbar vertebral bodies and the 4 intervertebral discs between L1 and L2, L2 and L3, L3 and L4, and L4 and L5; and the 10 facet joints between L1 and L2, L2 and L3, L3 and L4, L4 and L5, and L5 and S1.

### 2.2 Study population

CAMONA included 80 healthy individuals with low cardiovascular disease risk and 50 patients with angina pectoris with higher cardiovascular disease risk. The subjects were recruited from the blood bank at Odense University Hospital, Odense, Denmark, or via local advertisement. Individuals with no history of malignant diseases, immunodeficiency syndromes, autoimmune diseases, illicit drug use, alcohol abuse, major skeletal deformity or cardiovascular diseases were considered healthy and eligible for inclusion. We aimed for maximum variation between LBP and control groups in terms of pain but minimal variation in terms of age and sex. On the day of image acquisition, eligible individuals were asked to fill in the Standardized Nordic Musculoskeletal Questionnaire (Kuorinka et al., 1987). This is a 20‐item, self‐report patient questionnaire with questions relating to localization and intensity of spinal pain. This questionnaire is valid, reliable and responsive, and is widely used in epidemiological studies on neck and back pain. The LBP group was composed of individuals, who answered ‘yes’ to the question ‘have you had LBP in the past week?’ The control group comprised individuals, who reported (a) ‘once or twice’ or ‘never’ to the question ‘have you had LBP?', (b) ‘no’ to having ‘LBP within the past week’ and (c) ‘0’ to ‘LBP intensity today’. Matching by age was done allowing a maximum age difference of 3 years between LBPs and controls. As the majority of individuals did not report LBP pain (the prevalence of weekly LBP was only 18% among the 139 CAMONA subjects), it turned out that the maximal number of age and gender matches in the CAMONA cohort was 12 pairs. CAMONA was approved by the Danish National Committee on Health Research Ethics and registered at ClinicalTrials.gov (NCT01724749). The study was conducted in accordance with the Declaration of Helsinki and all study subjects provided written informed consent.

### 2.3 PET/CT protocol

The imaging protocol is described elsewhere (Blomberg, Thomassen, Takx, Hildebrandt, et al., 2014; Blomberg, Thomassen, Takx, Vilstrup, et al., 2014). FDG‐PET/CT imaging was performed on General Electric hybrid PET/CT systems of fairly comparable sensitivity (GE Discovery 690, VCT, RX and STE), such that seven subjects had their FDG and NaF scans on the same scanner, while the remaining 17 subjects were imaged on two different scanners. FDG PET/CT imaging started 180 min after intravenous injection of ~4.0 MBq/kg of FDG following an overnight fast of at least 6 h. The blood glucose concentration was determined to secure a value below 8 mmol/L before FDG injection. NaF‐PET/CT scans were performed 90 min after intravenous administration of 2.2 MBq/kg (max 400 MBq) of NaF. The estimated effective patient radiation doses were 6.5 and 6.0 mSv for the FDG‐ and NaF‐PET/CT scans, respectively. The subjects rested in a quiet and warm room after injection. Scans were acquired with 2.5 min per bed position. Correction of PET images for random, scattered coincidences, attenuation and anatomic directions was done by implanting transmission maps produced by a 64‐slice CT scan as follows (120 kV, 200 mA, 16 × 2.5 mm collimation, 0.5 s per rotation).

### 2.4 Image analysis

An automatic method to detect three volumes of interest (VOIs), that is, the lumbar bodies, facet joints and intervertebral discs, was used to analyse the images. The method was based on an organ segmentation tool capable of segmenting 100 different organs and bones in a cloud image processing platform named RECOMIA. The organ segmentation tool was a further developed version of the one presented by Trägårdh et al. (2020) and used a convolutional neural network (CNN) with a U‐Net structure (Çiçek et al., 2016). Before this study, the automated system was trained using 339 manually annotated CT images. In this study, a subset of the original 100 segmentation labels is used, namely the lumbar vertebra segmentations that are output as individual segmentations. Given the segmentation output from the segmentation tool, the VOIs were extracted using morphology. Initially, the lumbar bodies were segmented for each vertebra by eroding the initial segmentation at a distance of 10 mm. From the eroded vertebra, the largest component was then dilated, giving a segmentation of the vertebral body. To ensure that the entire vertebral body was included, the dilation distance was slightly larger than 10 mm in all directions, except towards the pedicle. The final segmentation of the vertebral body was taken as the overlap of the dilated segmentation and the initial segmentation of the vertebra.

To extract the facet joints, the contact area between two vertebrae was calculated as an initial step. This was done by dilating the posterior aspects of the vertebra between the vertebral body and the spinous process along the transverse axis and calculating the overlap between neighbouring vertebrae. The contact area was then dilated within the original vertebra segmentations to define the facet joint segmentation. For robustness, only the two largest components were kept and any parts too close to the centerline of the vertebra along the sagittal axis were removed. For the lowest situated facets, the contact area could not be used. Instead, the lowest part of the original vertebra segmentation was used as an initial segmentation. This was then dilated and refined similarly to the other facet joints. Each intervertebral disc was segmented utilizing the vertebral bodies above and below. The bodies were dilated along the transverse axis and the initial estimate of the intervertebral disc was taken as the overlap of the dilated vertebrae. To avoid including voxels to the sides of each vertebral body, two forbidden regions were defined by dilating the vertebral bodies in the transverse plane. The voxels in the forbidden regions were removed from the initial estimate, giving the final intervertebral disc segmentation.

**Figure 1:**
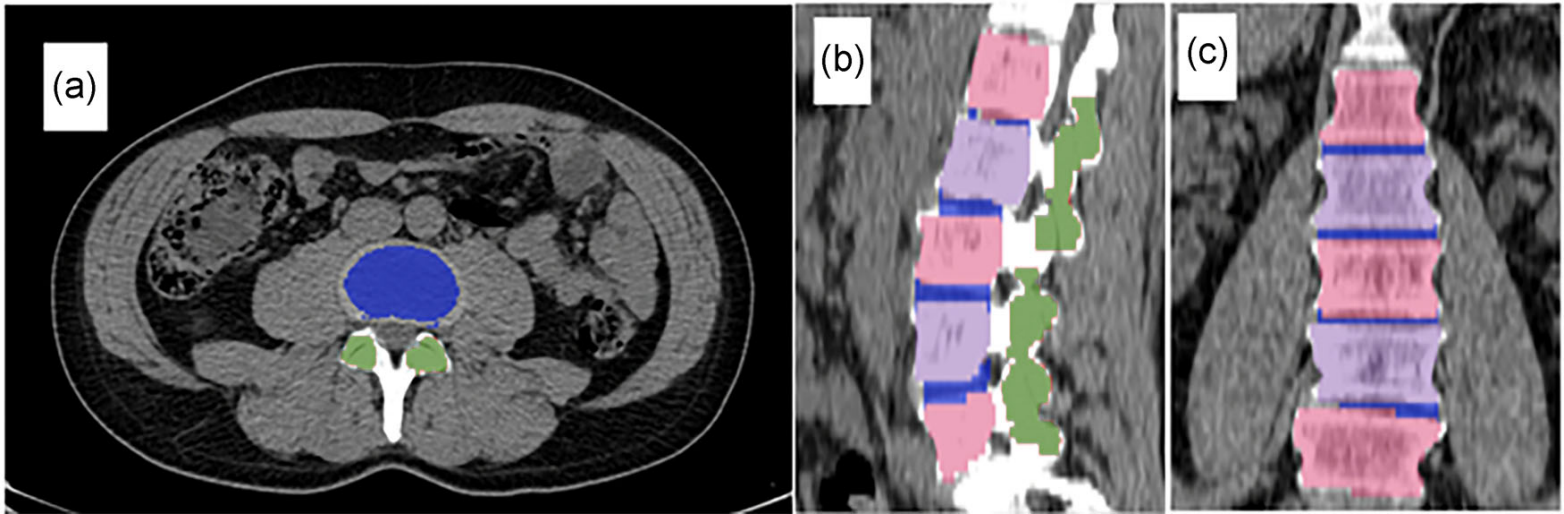
A sample of the semiautomatic segmentation shown in the computed tomography images in transaxial (a), sagittal (b) and coronal (c) planes. Vertebral bodies are shown with intermittent light blue and light red colours, facets with dark green colour and intervertebral discs with dark blue.

An example of the segmentation of lumbar vertebral bodies, facet joints and intervertebral discs appears in Figure 1. Finally, the segmented VOIs were reviewed by two image analysers (R. P. and A.  H. N. ‐F.) and modified if needed. The maximum, mean and total standardized uptake values (SUVmax, SUVmean and SUVtotal) for FDG and NaF, and same measure in Hounsfield unit (HUmean, HUmax and HUtotal) for CT in the three VOIs were measured and compared between the two groups. SUVmean was the average SUV of the entire VOI, SUVmax the highest SUV of all voxels in the VOI and SUVtotal was SUVmean multiplied by the VOI volume.

### 2.5 Statistical analysis

Descriptive statistics were expressed as frequency (percentage) and mean ± SD if differences followed a normal distribution and as median (minimum–maximum) otherwise. To compare quantitative data between matched groups, Student's paired *t* test was used, if the differences followed a normal distribution; alternatively, the nonparametric Wilcoxon matched‐pairs signed‐rank test was applied. The level of statistical significance was 5% and the Holm–Bonferroni method was applied to account for multiple testing (Holm, 1979). To this end, all *p* intended for inferential interpretation were sorted ascendingly and the smallest *p* was compared with the adjusted significance level of 0.05 m^−1^, with m statistical hypothesis tests considered. If the smallest *p* < 0.05 m^−1^, the respective null hypothesis was rejected and the second smallest *p* was subsequently compared with 0.05 m^−1^. If the smallest *p* was equal to or exceeded 0.05 m^−1^, the respective null hypothesis could not be rejected and the testing procedure ended with no statistical differences concluded. All statistical analyses were performed using SPSS 24.0 (SPSS Inc.).

## 3 RESULTS

Seven males and five females with a mean age of 45 years in each of the LBP and control groups underwent both FDG and NaF PET/CT imaging. The demographics of the two groups were not statistically different (Table 1) nor were their laboratory findings (not shown).

**Table 1:** Demographic information about the case and control groups

	Group		*p*
	Case (*n* = 12)	Control (*n* = 12)	
Age (years)	45 ± 14	45 ± 12	0.93
Gender	7 males, 5 females	7 males, 5 females	0.99
Weight (kg)	83.0 ± 12.8	76.3 ± 13.6	0.28
Height (cm)	174.3 ± 8.1	174.3 ± 10.3	0.98
Body mass index (kg/m^2^)	27.28 ± 3.43	24.91 ± 2.37	0.12

The median time gap between FDG and NaF studies was 1 week. The uptake of FDG was low in the lumbar vertebral bodies and facet joints of both groups in that SUVmean in these locations was below 2.0 and 1.0, respectively. In contrast, NaF uptake in these locations was about four to five times as high as the FDG uptake (Table 2). The general level of uptake of both tracers was low in the intervertebral discs of both groups as reflected by average SUVmean values < 1.0 for FDG and <3.0 for NaF. The VOI volumes did not differ between the groups.

**Table 2:** NaF and FDG uptake in different regions of the case and control groups

Variables		Group		Group difference (95% CI)	*p*
		Case (*n* = 12)	Control (*n* = 12)		
NaF					
Intervertebral discs	SUVmax	11.63 ± 3.29	9.45 ± 1.32	2.18 (0.15–4.21)	0.038
	SUVmean	2.75 (1.82–7.02)	2.55 (1.88–3.45)	0.64 (−0.35 to 1.64)	0.21^a^
	SUVtotal	76.73 ± 31.02	73.21 ± 22.60	3.51 (−16.88 to 23.91)	0.71
Facet joints	SUVmax	13.5 (7.23–33.98)	9.17 (8.25–17.69)	4.38 (1.08–7.68)	0.015^a^
	SUVmean	5.04 (3.53–7.8)	4.15 (3.42–7.21)	0.47 (−0.53 to 1.47)	0.41^a^
	SUVtotal	203 ± 48.60	196.92 ± 46.01	6.07 (−30.61 to 42.76)	0.72
Lumbar vertebral bodies	SUVmax	14.67 ± 4.17	12.72 ± 1.96	1.95 (−0.71 to 4.61)	0.13
	SUVmean	7.80 ± 1.98	7.29 ± 0.78	0.51 (−0.72 to 1.74)	0.38
	SUVtotal	1660.03 ± 503.91	1511.30 ± 205.81	148.74 (−101.88 to 399.35)	0.21
FDG					
Intervertebral discs	SUVmax	2.83 ± 1.04	2.26 ± 0.57	0.57 (−0.07 to 1.21)	0.08
	SUVmean	0.96 ± 0.34	0.69 ± 0.15	0.27 (0.05–0.49)	0.021
	SUVtotal	21.72 ± 7.76	19.82 ± 7.68	1.9 (−5.69 to 9.49)	0.59
Facet joints	SUVmax	2.47 (1.96–4.87)	2.84 (1.75–4.88)	−0.34 (−1.2 to 0.52)	0.35^a^
	SUVmean	0.99 ± 0.26	0.96 ± 0.20	0.03 (−0.19 to 0.26)	0.74
	SUVtotal	38.41 ± 4.79	42.18 ± 12.96	−3.77 (−13.12 to 5.58)	0.39
Lumbar vertebral bodies	SUVmax	3.77 ± 0.90	3.47 ± 1.06	0.3 (−0.6 to 1.2)	0.48
	SUVmean	1.75 ± 0.40	1.69 ± 0.40	0.06 (−0.3 to 0.43)	0.7
	SUVtotal	385.77 ± 137.84	359.15 ± 118.67	26.62 (−70.53 to 123.79)	0.55

Abbreviations: CI, confidence interval; FDG, fluorodeoxyglucose; NaF, sodium fluoride; SUV, standardized uptake value.

^a^ Wilcoxon matched‐pairs test was used.

When comparing the two groups, the FDG uptake was consistently slightly higher in LBP group than controls, except for higher SUVmax and SUVtotal in the facet joints of controls. FDG SUVmean in the intervertebral discs of LBP group (0.96 ± 0.34) exceeded that of controls (0.69 ± 0.15). All NaF uptake values were higher in cases than in controls and among these were a 23% higher SUVmax in the intervertebral discs and a 48% higher uptake in the facet joints (Table 2).

The differences in CT‐derived variables between the LBP and control group are shown in Table 3.

**Table 3:** CT findings in different regions of the case and control groups

Variables	Group		Group difference (95% CI)	*p*
	Case (*n* = 12)	Control (*n* = 12)		
Interevertebral discs				
HUmax	651.13 ± 100.20	606.85 ± 155.77	44.28 (−67.54 to 156.11)	0.4
HUmean	121.72 ± 13.50	117.58 ± 10.13	4.15 (−6.6 to 14.89)	0.41
HUtotal	3165.69 ± 1181.62	3339.46 ± 922.39	−173.77 (−190.39 to 562.85)	0.61
Facet joints				
HUmax	1220.69 ± 65.24	1248.34 ± 51.10	−27.66 (−70.74 to 15.43)	0.18
HUmean	536.28 ± 114.54	498.93 ± 79.76	37.36 (−27.2 to 101.91)	0.23
HUtotal	21926.61 ± 5984.90	22191.24 ± 6600.23	−264.63 (−5397.5 to 4868.24)	0.91
Lumbar vertebral bodies				
HUmax	1039.48 ± 109.10	1026.57 ± 90.90	12.9 (−77.67 to 103.47)	0.76
HUmean	238.84 ± 56.93	235.39 ± 33.95	3.45 (−26.57 to 33.47)	0.8
HUtotal	50974.52 ± 14722.54	49505.45 ± 10925.34	1469.07(−6255.32 to 9193.47)	0.68

Abbreviations: CI, confidence interval; CT, computed tomography; HU, Hounsfield unit.

None of the differences were statistically significant after Holm–Bonferroni correction for the 27 comparisons in Tables 2 and 3, as the smallest *p* of 0.015 exceeded the adjusted significance level of 0.05/27 = 0.002 in the first step of the testing procedure.

## 4 DISCUSSION

### 4.1 Principal findings

Comparing FDG and NaF uptake in lumbar vertebral bodies, facet joints and intervertebral discs in the LBP group with uptake in the age‐ and gender‐matched control group, we found higher FDG uptake in most locations of the case group, with higher FDG SUVmean in the intervertebral discs and higher NaF uptake in all locations including higher NaF SUVmean and SUVmax in facet joints and the intervertebral discs of individuals with LBP (Table 2).

### 4.2 Strengths and weaknesses of the study

We were able to compare two different metabolic processes in a case and comparable control group, a setup that even with such relatively small groups is a rarity in LBP research. A further strength was the utilization of an AI‐based algorithm for segmentation of the VOIs. In our material, small manual modifications were made in <10% of AI‐segmented regions. Besides faster processing, the CNN approach has an inborn 100% repeatability at reanalysis of the same set of PET/CT scans. Due to use of different scanners and not the same scanner on both occasions in all subjects, we cannot guarantee that all measured values are absolutely correct and representative for the subjects with and without LBP; this has to be validated in larger prospective studies using the same or very similar PET/CT scanners in all subjects. However, CNN‐based image analysis has been shown to be even more reproducible than manual segmentation, when scans from different scanners are processed (Belal et al., 2019), and our findings indicate that AI‐based segmentation of relevant lumbar spine structures are indeed feasible.

This study was explorative and the relatively small size of our two groups was a limitation, as it may cause under‐ or overestimation of observed effects. Another limitation was the relatively low spatial resolution of PET and the fact that CT is inferior to MRI in visualizing soft tissue (Beattie & Meyers, 1998). These circumstances mostly affect segmentation of intervertebral discs due to their soft tissue nature. In addition, due to the lower spatial resolution of PET, it may be difficult with PET alone to discriminate between high tracer uptake in a target organ and an adjacent structure with similar or even higher uptake. This was seen in our material with the vertebral bodies, which have an excessively high NaF uptake compared with nonbony structures.

### 4.3 Comparison of finding of the study in relation to other studies

Unlike most other studies reporting later occurring macroscopic structural changes, we searched for nociceptive sources of LBP at the early molecular level. FDG‐PET has a long history of investigation of musculoskeletal disorders, especially when aiming to differentiate malignancies and infectious processes from degenerative processes. The latter is perhaps one of the most prevalent causes of LBP (Chou et al., 2011) and a systematic review has demonstrated that changes such as intervertebral disc bulging, degeneration and extrusion have a reasonably strong association with LBP (Brinjikji, Luetmer, et al., 2015). Degenerative changes are shown to have lower FDG uptake compared with malignancies and infectious processes (Ohtori et al., 2010). However, the degree of FDG uptake is a spectrum rather than a dichotomous feature, meaning that degenerative changes may have FDG uptake to some extent, but lower than conditions with a higher inflammatory profile. It has been shown that the severity of degenerative changes correlates with spinal FDG uptake in individuals with known or suspected malignancy (Rosen et al., 2006). Similarly, in the current study, individuals in the case group had higher FDG uptake in the intervertebral discs, demonstrating higher inflammation than in the control group. A hypothesis regarding inflammation initiation in intervertebral discs relies on endogenous factors, for example, breakdown products of extracellular matrix and crystals, such as hydroxyapatite and calcium pyrophosphate dihydrate (Molinos et al., 2015). Phagocytosis of crystals could be a trigger for inflammation leading to monocyte gathering (Marom et al., 2007). Interestingly, the findings of the current study support this hypothesis, because individuals in the case group had a higher NaF uptake, which mainly targets calcium depositions, in the intervertebral discs. Other studies have shown that degenerative changes are a consequence of inflammation and the associated oxidative stress in animal models (Chen et al., 2017).

Degenerative changes in the facet joints are found to be associated with LBP (Suri, Dharamsi, et al., 2013; Suri, Hunter, et al., 2013). In our sample, we found a tendency for higher uptake of NaF in the LBP group compared with controls in facet joints and intervertebral discs. NaF‐PET examines bone turnover, but with a higher resolution than former conventional modalities (Raynor et al., 2016). Previous studies have also suggested that high NaF uptake is associated with facetogenic pain and facet deformities (Gamie & El‐Maghraby, 2008; Mabray et al., 2016). In a study reporting NaF uptake in 67 patients, some of which had undergone discectomy, laminectomy or lumbar fusion, routine X‐ray, CT or MRI failed to find the cause of LBP, whereas NaF uptake in facet joints was abnormal in the majority of individuals (56/67; Gamie & El‐Maghraby, 2008). The image analysis was qualitative and the study individuals were divided into NaF positives and NaF negatives, that is, a simplification that precludes meaningful comparison with our quantitative results.

### 4.4 Possible mechanisms and implications

The NaF uptake in the facet joints of cases was higher than in control individuals, but the difference in NaF uptake in the facets was not accompanied by a similar FDG uptake difference. An explanation could be that consequent degenerative and rebuild changes that take place after an initial triggering inflammation process may go on long after the inflammation has resolved, or that active calcification may take place in the facet joints independent of inflammation. The latter scenario is supported by some studies reporting low efficacy of intraarticular facet corticosteroid injection in terms of pain relief (Barnsley et al., 1994; Carette et al., 1991). The relationship between initiation of degenerative changes and inflammatory cytokines is relatively well established (Igarashi et al., 2004; Kim et al., 2015; Xu et al., 2013). However, the lack of difference in FDG and NaF uptake in the lumbar vertebral bodies may suggest that one or both processes that the tracers mirror are not involved in generating pain. On the whole, the vertebral bodies are complex structures consisting of the bone marrow, vasculature and bone, all of which have different metabolic profiles, and this together with multiple other factors makes interpretation of the significance of the relative tracer uptake in the lumbar region challenging.

The pattern of lumbar FDG and NaF uptake that we observed in individuals with and without LBP indicate that it may be worthwhile to further explore the use of PET/CT or PET/MRI imaging in cases of LBP. Thus, molecular aspects and the potential of imaging initial phases of LBP development appear to call for further PET studies of LBP, although it is far too early to suggest a more substantial role of PET imaging in the diagnosis and management of patients with LBP.

### 4.5 Unanswered questions and future research

Although our study may suggest an explorative role of PET in characterizing patients with LBP, it is still not known if and how inflammation and microcalcification correlate in different regions of the low back, or which process is horse or carriage, provided there is a causal connection between them. Future research, including longitudinal studies with PET/CT and PET/MRI in larger and more well‐defined study groups are needed to investigate the temporal association between inflammation and microcalcification in different low back regions.

## 5 CONCLUSION

Inflammation and degenerative changes, mirrored by increase in FDG and NaF uptake in the facet joints and intervertebral discs of individuals with LBP suggest an explorative role of PET imaging in this disorder, in particular because AI‐based processing, which facilitates and speeds up the analysis, appears feasible. However, further studies applying molecular imaging in more well‐defined, larger and longitudinal studies are warranted to elucidate the potential clinical impact of these modalities in the diagnosis and management of patients with LBP.

## CONFLICTS OF INTEREST

The authors declare no conflicts of interest.

## Data Availability

The anonymized data that support the findings of this study are available from the corresponding author, Reza Piri, upon reasonable request.
